# Frequency and clinical characteristics of hypophysitis and hypopituitarism in patients undergoing immunotherapy – A systematic review

**DOI:** 10.3389/fendo.2023.1091185

**Published:** 2023-02-15

**Authors:** Juliana Prudêncio Jacques, Luciana Pinto Valadares, Adriana Castelo Moura, Maria Regina Fernandes Oliveira, Luciana Ansaneli Naves

**Affiliations:** ^1^ Faculty of Health Science, University of Brasilia, Brasilia, Brazil; ^2^ Department of Internal Medicine and Endocrinology, Sarah Network of Rehabilitation Hospitals, Brasília, Brazil; ^3^ Department of Oncology, Hospital Universitário de Brasília, Brasília, Brazil; ^4^ Faculty of Medicine, University of Brasília, Brasília, Brazil

**Keywords:** immunotherapy, hypophysitis, hypopituitarism, immune check point inhibitors (ICI), cancer, pitutary

## Abstract

**Objective:**

To describe the frequency of hypophysitis and hypopituitarism in cancer patients who are undergoing antineoplastic treatment with immunotherapy, as well as to describe the clinical, epidemiological, and demographic characteristics of these patients.

**Methods:**

A systematic search of the literature in PubMed, Embase, Web of Science, ClinicalTrials.gov and Cochrane Controlled Register of Trials took place on May 8 and 9, 2020. Randomized and nonrandomized clinical trials, cohort studies, case-control studies, case series and case reports were included.

**Results:**

A total of 239 articles were obtained, in which 963 cases of hypophysitis and 128 cases of hypopituitarism were found in a treated population of 30,014 individuals (3.20% and 0.42% of the evaluated population, respectively). In the cohort studies, the incidence of hypophysitis and hypopituitarism ranged from 0% to 27.59% and from 0% to 17.86%, respectively. In the non-randomized clinical trials, the incidence of hypophysitis and hypopituitarism ranged from 0% to 25% and from 0% to 14.67%, and in randomized clinical trials from 0% to 16.2% and from 0% to 33.33%. The most common hormonal changes were in the corticotrophic, thyrotrophic and gonadotrophic axes. The main magnetic resonance imaging (MRI) findings were enlargement of the pituitary gland and enhanced contrast uptake. The main symptoms presented by patients with hypophysitis were fatigue and headache.

**Conclusion:**

The present review reported a frequency of hypophysitis and hypopituitarism of 3.20% and 0.42%, respectively, in the evaluated population. The clinical-epidemiological characteristics of patients affected by hypophysitis were also described.

**Systematic review registration:**

https://www.crd.york.ac.uk/prospero/, identifier CRD42020175864.

## Introduction

According to the World Health Organization (WHO), cancer is the second leading cause of death globally and accounted for nearly 10 million deaths in 2020 (1). Cancer treatments have experienced substantial advances in recent years, with immunotherapy consolidating itself as one of the antineoplastic therapeutic pillars in the last decade (2). Within immunotherapy, medications that target immune checkpoints have emerged as treatment options with the possibility of lasting responses and long-term benefits (2, 3). These immune checkpoint inhibitors (ICIs) enhance the anti-tumor action of the immune system by blocking negative regulators of T cell function, present in both immune and tumor cells (4).

Among immune checkpoints, CTLA-4 and PD-1 are the most avidly studied (4). The use of ICIs has led to adverse effects resulting from the increase in autoimmunity, which are called immune-related adverse events (irAEs). Among the endocrine-related irAEs, hypophysitis is one of the most common. The inflammatory condition may be transient or permanent, resulting in hypopituitarism in the latter.

The objective of this systematic review is to describe the frequency of hypophysitis and hypopituitarism in cancer patients who are undergoing antineoplastic treatment with ICIs and to describe the clinical, epidemiological, and demographic characteristics of those patients affected by hypophysitis.

## Method

The present systematic review was reported according to the Preferred Reporting Items of Systematic Reviews (PRISMA) statement. and registered in the Prospective Register the Systematic Reviews (PROSPERO) database (5).

### Search strategy and study selection

We carried out a systematic review of the literature in multiple databases, including Medline (Pubmed), Embase, Web of Science, ClinicalTrials.gov, CENTRAL (Cochrane Controlled Register of Trials) and gray literature. A manual search for references in relevant articles and in other reviews was also carried out. The search took place on May 8 and 9, 2020.

We used the Boolean operators “AND” and “OR” to combine the terms of the search strategy: “Hypophysitis”, “Hypopituitarism”, “GH deficiency”, “TSH deficiency”, “ACTH deficiency”, “Hypogonadism”, “ipilimumab”, “MDX-010”, “tremelimumab”, “CP-675206”, “nivolumab”, “BMS-963558”, “pembrolizumab”, “MK-3475”, “atezolizumab”, “MPDL3280A”, “avelumab”, “MSB0010718C”, “durvalumab”, “MEDI4736”, “PD-1”, “PDL-1”, “CTLA-4”, “immune checkpoint inhibitors.”

The evaluation included randomized and nonrandomized clinical trials, cohort studies, case-control studies, descriptive studies, case series and case reports. The target population consisted of cancer patients undergoing treatment with immunotherapy. Only complete articles available in Portuguese or English were considered, in addition to results published in ClinicalTrials.gov. Papers published only in conference proceedings were excluded. There was no restriction regarding the year of publication.

Four reviewers (J.P.J, L.P.V, L.A.N and A.C.M) participated in the selection and evaluation of the studies. In the first phase, J.P.J. and L.P.V., independently and blindly, carried out the initial selection of studies based on titles and abstracts, applying the inclusion and exclusion criteria after excluding duplicate works. When there were divergences, these were discussed, and the discrepancies were resolved by consensus or, when not reached, a third researcher (L.A.N) made the assessment.

For the second phase, works were evaluated in their entirety. As the first reviewer, J.P.J. made the evaluation of all articles that were selected for the second phase. Studies were divided into two groups: (i) articles from case reports, case series and cohort studies with description of cases of hypophysitis were evaluated by L.P.V.; and (ii) the remaining cohort studies, the non-randomized clinical trials (NRCTs) and the randomized clinical trials (RCTs) were evaluated by A.C.M. The evaluation of the articles was carried out in a paired and independent manner.

### Data collection and quality assessment

Data from the selected articles were independently extracted by each reviewer, and discrepancies were resolved by consensus or with the assessment of the third researcher.

Information collected from each study were, when available: title of the study, author(s) and location of the study, year of publication, sample size, study design, age and gender of the patient(s), medication and dose used, duration of treatment, number of patients with hypophysitis and hypopituitarism, time until the appearance of the pituitary alteration, symptoms at diagnosis, and the hormonal and radiological alterations presented.

The methodological quality and the risk of bias of the articles were assessed according to the Joanna Briggs Institute Critical Appraisal Checklist (6).

### Data analysis

For the description, presentation and data analysis, the studies were divided into: (i) studies for the analysis of the incidence of hypophysitis and hypopituitarism; and (ii) studies that aim to describe the clinical and epidemiological characteristics of patients affected by hypophysitis, according to the geographic region of the study, making a comparison among continents, countries, and other characteristics. The data analysis was done using Excel.

## Results

### Study selection and characteristics

**Figure 1:**
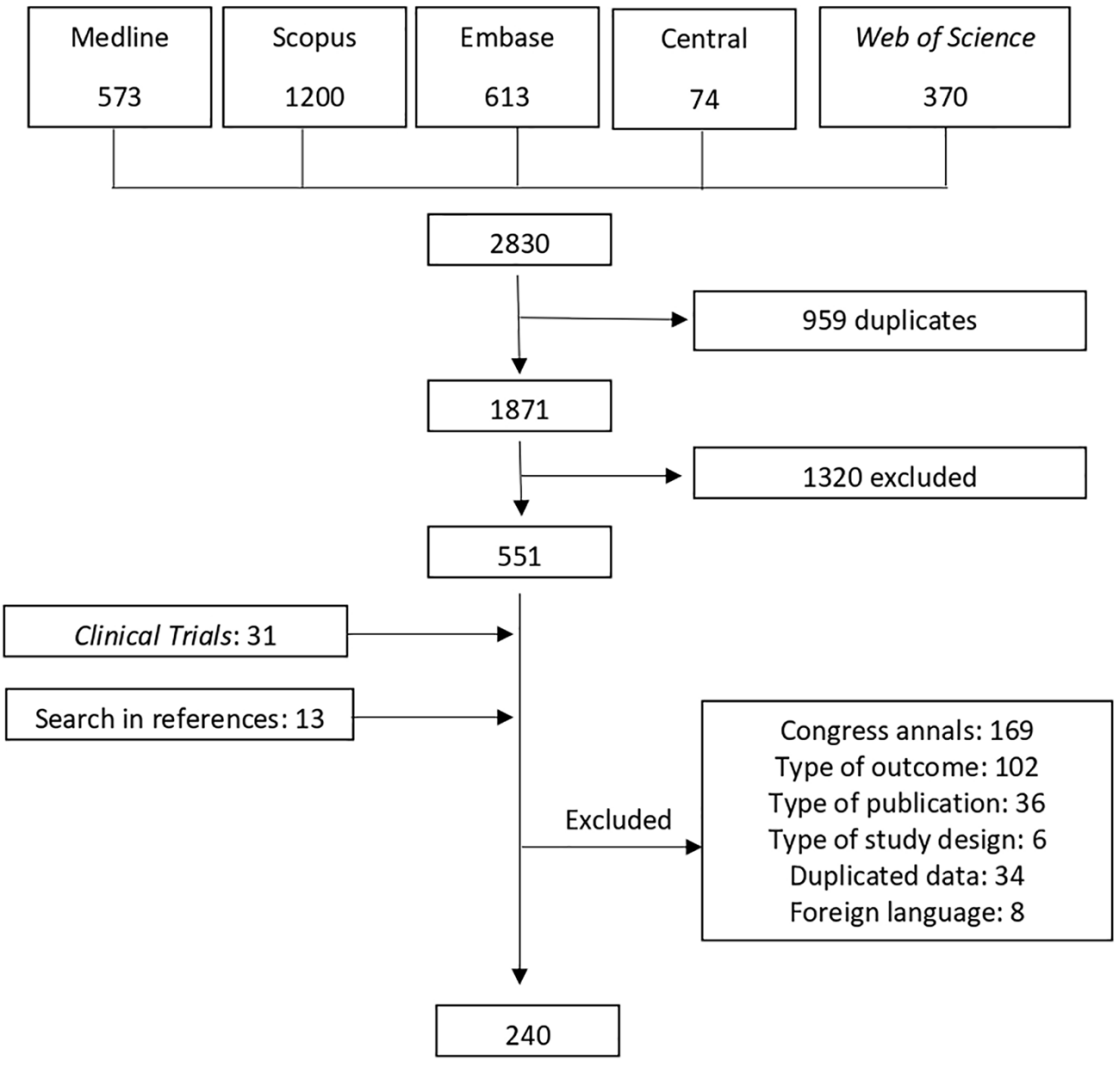
Flow diagram of the stages of study selection; NRCT, Non-randomized clinical trial; RCT, Randomized clinical trial.

The search strategy identified a total of 2,830 articles. After removing duplicates, 1,871 papers remained, and their titles and abstracts were evaluated for suitability. Following this initial evaluation, a selection of 551 articles were fully evaluated. In phase 2, the selection process led to the exclusion of 356 studies. Thirteen additional studies and 31 clinical trials were included as identified from the references of the retrieved articles. Therefore, 239 articles were retained for the final analysis of the systematic review. The search strategy is outlined in **Figure 1**.

### Synthesis of results

The 239 studies included in the final analysis comprised a total of 30,014 patients. All 239 included studies were published in English or Portuguese between 2002 and 2018, but most of them were carried out in the period from 2011 to 2015. The studies were conducted in 19 different countries: Australia, Belgium, Brazil, Canada, China, Czech Republic, France, Germany, Greece, Israel, Italy, Japan, Portugal, Romania, Spain, Taiwan, The Netherlands, United Kingdom, United States of America (USA), however, multicenter studies were the majority, encompassing 82.5% of RCTs.

### Assessment of incidence of hypophysitis and hypopituitarism

To assess the incidence of hypophysitis and hypopituitarism, data from descriptive and analytical cohort studies, and from clinical non-randomized and randomized studies was computed. In articles with two or more arms, each arm was computed separately. Considering all arms of the studies, 963 cases of hypophysitis and 128 cases of hypopituitarism were identified in a treated population of 30,014 individuals (3,20% and 0.42%, respectively, of the evaluated population.) There was no meaningful difference in the incidence of hypophysitis and hypopituitarism between the types of studies considered (cohort, RCTs, NRCTs), regarding the minimum and maximum values ​​presented in these studies. The minimum and maximum incidences of hypophysitis found in each type of study evaluated were, in cohort studies 0% (7–11) and 27,59% (12), in NRCT 0% (12, 13) and 25% (14) and in RCT 0% (15–18) and 16,21% (19). For hypopituitarism, the incidences varied from 0% to 17,86% in cohort studies, from 0% to 14,67% in NRCT and 0% to 33% in RCT.

Comparing the incidence of hypophysitis in combination immunotherapy with monotherapy, there was a higher frequency in combination therapy. In RCTs, it ranged from 1.61% to 25.71%, while in monotherapy this incidence varied from 0% to 16.21%.

In the present review, the incidence of hypophysitis ranged from 1.3% to 6.17% in RCTs, from 0% to 6.76% in cohort studies and from 0% to 7.26% in NRCTs when patients were treated with CTLA-4 inhibitors. In the treatment with PD(L)-1 inhibitors the incidence of hypophysitis varied from 0% to 0.67% in RCTs, from 0% to 2.10% in cohort studies and from 0% to 2.28% in NRCTs.

As shown in **Tables 1**–**3**, melanoma was the most treated cancer in the three study groups, reaching 63,5% in the cohort studies, followed by lung cancer, which reached 22,5% in RCTs. Regarding the type of immunotherapy used in the treatment, ipilimumab was the most studied, used in 51,2% of NRCTs, 37,8% in cohort studies and 37.5% of RCTs. Nivolumab was the second most used drug in cohort studies (12,1%) and pembrolizumab the second most used in NRCTs (22%) and RCTs (20%).

**Table 1:** Distribution of cohort studies according to the type of cancer studied, place of study, medication used and year of study initiation.

Type of cancer	Melanoma 47 (63,5%)	
	Lung 8 (10,8%)	
	Renal 5 (6,7%)	
	Multiple neoplasias 14 (19%)	
		
Study site	USA 24 (32,4%)	Mexico 1 (1,3%)
	Canada 2 (2,7%)	Australia 4 (5,4%)
	Europe 31 (41,9)	Multicenter 4 (5,4%)
	Asia 8 (10,8%)	
	
Medication	Ipilimumab 28 (37,8%)	
	Nivolumab 9 (12,1%)	
	Anti-PD-1 5 (6,8%)	
	Pembrolizumab 4 (5,5%)	
	Multiple 28 (37,8%)	
	
Year of study initiation	2002 1 (1,3%)	2012 4 (5,5%)
	2005 2 (2,7%)	2013 7 (9,5%)
	2006 2 (2,7%)	2014 6 (8,1%)
	2007 2 (2,7%)	2015 11(14,8%)
	2008 4 (5,5%)	2016 5 (6,7%)
	2009 4 (5,5%)	2017 2 (2,7%)
	2010 6 (8,1%)	2018 2 (2,7%)
	2011 7(9,4%)	NR 9 (12,1%)

NR, Not reported; USA, United States of America.

**Table 2:** Distribution of non-randomized clinical trials according to the type of cancer studied, place of study, medication used and year of study initiation.

Type of cancer	Melanoma 23 (56%)	Ovary 2 (4,9%)
	Lung 6 (14,6%)	Renal 1 (2,4%)
	Prostate 4 (9,8%)	Other Neoplasias 3 (7,3%)
	Urothelial 2 (4,9%)	
	
Study site	USA 18 (44%)	United Kingdom 1 (2,4%)
	Multicenter 15 (36,5%)	Belgium 1 (2,4%)
	Japan 4 (9,8%)	Germany 1 (2,4%)
	Holland 1 (2,4%)	
	
Medication	Ipilimumab 21 (51,2%)	Durvalumab 1 (2,4%)
	Pembrolizumab 9 (22%)	Combination therapy 1 (2,4%)
	Nivolumab 3 (7,3%)	Not specified 4 (9,8%)
	Tremelimumab 2 (4,9%)	
	
Year of study initiation	2002 1 (2,4%)	2012 5 (12,1%)
	2004 2 (4,9%)	2013 2 (4,9%)
	2005 2 (4,9%)	2014 6 (14,6%)
	2008 1 (2,4%)	2015 5 (12,1%)
	2009 4 (9,8%)	2016 4 (9,8%)
	2010 3 (7,3%)	2017 2 (4,9%)
	2011 2 (4,9%)	NR 2 (4,9%)

NR, Not reported; USA, United States of America.

**Table 3:** Distribution of randomized clinical trials according to the type of cancer studied, location of the study, medication used and year of study initiation.

Type of cancer	Melanoma 15 (37,5%)	Multiple neoplasias 1 (2,5%)
	Lung 9 (22,5%)	Breast 1 (2,5%)
	Head and neck 3 (7,5%)	Ovary 1 (2,5%)
	Gastric 3 (7,5%)	Pancreas 1 (2,5%)
	Prostate 3 (7,5%)	Renal 1 (2,5%)
	Mesothelioma 2 (5%)	
	
Study site	Multicenter 33 (82,5%)	
	USA 5 (12,5%)	
	Australia 1 (2,5%)	
	France 1 (2,5%)	
	
Medication	Ipilimumab 15 (37,5%)	Tremelimumab 2 (5%)
	Multiple 9 (22,5%)	Avelumab 1 (2,5%)
	Pembrolizumab 8 (20%)	Durvalumab 1 (2,5%)
	Nivolumab 3 (7,5%)	Combination therapy 1 (2,5%)
		
Year of study initiation	2004 1 (2,5%)	2012 5 (12,5%)
	2005 1 (2,5%)	2013 8 (20%)
	2008 3 (7,5%)	2014 9 (22,5%)
	2010 1 (2,5%)	2015 4 (10%)
	2011 2 (5%)	2016 6 (15%)

Abbreviations: USA: United States of America

### Evaluation of clinical and epidemiological characteristics

To assess the clinical and epidemiological characteristics of patients with hypophysitis, 78 case reports, six case series and 13 cohort studies were evaluated; a total of 376 patients were analyzed. The cases were grouped by country and, subsequently, by continent.

Of the total of patients described, 255 (67.81%) were male and 121 (32.19%) were female. The mean average age was 60.85 years. The time between the start of immunobiological therapy and the onset of hypophysitis symptoms was 124.93 days (17.8 weeks). Considering the hormonal deficiencies described, isolated ACTH deficiency was found in 35 cases and diabetes insipidus was diagnosed in 5 cases. In the 355 cases where MRI was performed, no cases of pituitary metastasis as a cause of hypopituitarism were described. The dosage of antibodies related to the hypophysis was not overall performed as part of the diagnosis.

It was not possible to assess the number of patients who recovered their pituitary function in all the cases analyzed, as this data was not always available. Of these patients, 17 resulted with the recovery of their corticotropic axis and did not require chronic glucocorticoid replacement. On the cases where follow-up was described, 15 deaths were reported.

The symptoms most commonly presented at the diagnosis of hypophysitis were fatigue/malaise (57,97%), followed by headache (46,8%) and nausea (24,2%). Fever was reported in 6 cases (1,59%).

For the epidemiological characteristics evaluated, the cases were grouped by country and, subsequently, by continent.

In North America, 36 studies and 220 patients with hypophysitis were found, while in South America our systematic search found only one study (20) with one reported case. In Asia, 62 patients were found, distributed between seven studies, and in Europe, 22 studies with 68 patients described. Oceania is represented by six studies with 25 reported cases.

Proportionally, the largest number of studies, 36, corresponding to 37%, were done in North America, followed by Asia with 32 studies (33%). However, in number of described patients, Europe surpasses Asia with 68 reported cases (18%).

The main information collected from the studies grouped by continents is detailed in **Tables 4**, **5**.

**Table 4:** Information of the case studies, case series and cohort studies describing individual cases, grouped by continent – Part 1.

Continent	North America	South America	Europe	Asia	Oceania
Nunber of patients	220	1	68	62	25
Gender(F/M)	70F/150M	M	20F/48M	26F/36M	5F/20M
Median age^(a)^	56 to 62,5	60	49 to 70	57,5 to 66,5	63
Cancer: prevalence order	1° Melanoma 2° Lung, Renal 3° Prostate	Melanoma	1° Melanoma 2° Lung 3° Others	1° Lung 2° Melanoma 3° Renal 4° Others	Melanoma
Treatment: prevalence order	1° Ipilimumab 2° Combination 3° Nivolumab 4° Others	Ipilimumab	1° Ipilimumab 2° Nivolumab 3° Combination, Pembrolizumab 4° Others	1° Nivolumab 2° Combination 3° Pembrolizumab 4° Others	1° Ipilimumab 2° Combination, Pembrolizumab
Main symptoms – prevalence order	1° Headache 2° Fatigue 3° Nausea 4° Others	1° Fatigue 2° Headache	1° Headache 2° Fatigue 3° Nausea 4° Others	1° Fatigue 2^a^ Anorexia 3° Nausea 4° Others	1° Fatigue, Headache 2° Nausea 3° Lethargy 4° Others

F, Female; M, Male; (a) The values presented are the lowest and highest median ages per country of the corresponding continent.

**Table 5:** Information of the case studies, case series and cohort studies describing individual cases, grouped by continent – Part 2.

Continent	North America	South America	Europe	Asia	Oceania
Image, main alterations – prevalence order	1° Enlargement 2° Enhancement 3° Normal 4° Thickening pituitary stalk 5° Others	Enlargement	1° Enlargement 2° Enhancement 3° Normal 4° Others	1° Normal 2° Alteration in the contour of the adenohypophysis 3° Enlargement 4° Enhancement 5° Others	1° Enlargement 2° Enhancement 3° Normal 4° Others
Main hormonal changes – prevalence order	1° AI 2° Hypothyroidism 3° Hypogonadism 4° PanHipo 5° HipoNa 6° PRL low 7° GH deficiency 8° PRL high	1° AI 2°Hypothyroidism	1° AI 2° Hypogonadism 3° Panhipo 4° Hypothyroidism 5° Others	1° AI 2° Hypogonadism 3° Hypothyroidism	1° AI 2° Hypothyroidism 3° Hypogonadism 4° GH deficiency

AI, Adrenal insufficiency; PanHipo, panhypopituitarism; HipoNa, hyponatremia; PRL, Prolactin; GH, Growth hormone.

### Quality assessment of the reviewed studies

The quality assessment of the case report studies revealed 35 studies (44.87%) with a low risk of bias, followed by 22 studies (28.21%) with moderate risk and 21 (26.96%) with high risk. Three case series presented high risk of bias, reaching 50% of the total cases. Most of the descriptive and analytical cohort studies had a low risk of bias, 83.33% and 63.15%, respectively. The majority of NRCTs (91.89%) also had a low risk of bias. For the RCTs, 16 studies (53.3%) had moderate risk and 13 studies (43.33%) had low risk of bias.

## Discussion

Cancer remains a major health problem around the world, and new therapies are constantly being studied. Immunotherapies, including checkpoint inhibitors, offer promising new treatment options and changed the treatment scenario and prognosis for several tumors. Hence, it is essential to understand the adverse events related to these drugs. So far, we conducted a systematic review assessing the incidence of endocrine-related adverse events, hypophysitis and hypopituitarism, in cancer patients.

Hypophysitis due to antineoplastic treatment is the most prevalent endocrinopathy in patients treated with CTLA-4 inhibitors, such as ipilimumab (21–24). A difference between hypophysitis of autoimmune origin and secondary to treatment with CTLA-4 inhibitors, is that the former is more frequent among women, while the latter is more frequent in men (20, 21, 23, 25).

The hormonal change generally observed in primary hypophysitis is similar to that observed in secondary to ICI treatment hypophysitis. That is, the order of appearance of changes in the function of pituitary cells, which occur at different frequencies, presents the same pattern. In both primary and secondary hypophysitis, the hormonal deficiencies follow, in most cases, the sequence: ACTH> TSH> LH/FSH> PRL> GH (25, 26).

Unlike other systematic reviews, which were limited to evaluating only clinical trials (250), we included case reports, case series and cohort studies. The aim of this broad selection was not only to increase the sensitivity of the search results, but also to obtain the clinical and epidemiological characteristics of the patients, information that was not normally included in the clinical trials included in other reviews (24, 27–30).

Comparing the incidence found in the present review with results in the literature, such as in the systematic review carried out by Lu et al. (27) that evaluated the pituitary-adrenal dysfunction associated with the use of immunotherapy, similar outcomes were observed. In their review, only RCTs were included, resulting in a total of 11,893 individuals, and the authors found an incidence of hypophysitis of 3.25% (95% CI: 2.15% -4.51%), which is within the numbers obtained in the present review for RCTs, which were from 0% to 16.21%.

When the variation in incidences was evaluated, all three study types presented the lowest incidence of hypophysitis as 0%. These studies without any reports of hypophysitis presented cases of hypopituitarism as an adverse event. Because hypopituitarism is, in these cases, due to pituitary inflammation, it can be inferred that hypophysitis cases were not diagnosed in the acute phase of the disease and were only identified after the installation of hormonal deficiencies, characterizing hypopituitarism as a late complication of pituitary impairment.

Additionally, most studies evaluated did not report cases of hypopituitarism. A hypothesis raised is that in these studies the evaluation was carried out in a follow-up inferior to that necessary for the clinical recognition of this condition.

Concerning the works with the highest incidence of hypophysitis in the cohort studies and NRCTs, both studies were small, 29 patients in the cohort (31) and 28 patients in the NRCT (19). The referred cohort study consisted of a historical cohort of melanoma patients who received ipilimumab 3mg/kg. The NRCT also followed patients who were treated with ipilimumab in a staggered dose, ranging from 0.3mg/kg to 5mg/kg. Hypophysitis was diagnosed when patients were using the highest dose. Also related to the highest dose of ipilimumab is the randomized clinical trial (32) that presents the highest incidence of hypophysitis. This clinical trial followed patients undergoing treatment with high doses of the medication, 10mg/kg, which may partly justify the high number of reported events. Luke et al. (33) performed a retrospective analysis of 39 patients with uveal melanoma from four centers in Europe and the USA. They demonstrated that irAEs were more frequent in patients who used the 10mg/kg dose of ipilimumab, when compared to those receiving the 3 mg/kg dose. Corroborating this finding, a double-blind, phase III multicenter study by Ascierto et al. (34), with 727 patients, also found an increase in adverse events in patients receiving the highest dose of the medication.

In the present study we observed that combination therapy presents a greater risk of leading to the appearance of pituitary changes such as hypophysitis, when compared to monotherapy, which is in accordance with the findings in the literature review. Barroso-Sousa et al. (24), found an incidence of hypophysitis for combination therapy of 6.4%, and also observed that patients who received combination therapy were significantly more likely to be affected with hypophysitis (OR, 2.2; 95% CI, 1, 39-3.6; P <0.001). corroborates results in the literature

Regarding the type of ICI used in the treatment, as well as the present review, previous work has also showed differences in the frequency of hypophysitis according to the mechanism of action. With CTLA-4 inhibitors, the overall incidence obtained in meta-analyses ranged from 3.2% to 4.53% (24, 27, 28). For PD(L)-1 inhibitors, the variation was from 0.3% to 0.5% (27, 29, 30). Xu et al. (28), for example, observed hypophysitis in 3.3% of patients treated with anti-CLTA-4. Barroso-Sousa et al. (24) showed an incidence of 3.2% for anti-CTLA-4 inhibitors, 0.4% for PD-1 inhibitors and less than 0.1% for PD-L1 inhibitors.

The difference between the frequency of pituitary involvement by anti-CTLA-4 and anti-PD-1 can be explained by the broader action of anti-CTLA-4, which affects the immune cycle in the initial stages of its activation. This action differs from that of anti-PD1 and anti-PD-L1, which act in tumor microenvironment and in circulation (35). Other explanations may be the presence of pituitary CTLA-4 receptors, complement deposition in the pituitary with local cell infiltration, and the formation of antibodies against pituitary cells (1, 27, 36).

For the assessment of clinical and epidemiological characteristics, 376 cases were extracted. To our knowledge, this is the largest compilation of cases on hypophysitis carried out to date. There were differences among the number of cases described in each country. The USA, for instance, encompasses the greatest number of studies performed and most of the patients analyzed. One of the hypotheses for these differences may be related to the different degree of resources available to academic research in each country. However, a more likely general explanation is the underdiagnosis of hypophysitis since the symptoms may be confused with those of the underlying pathologies. Another hypothesis is related to the date of approval of immunotherapies by the regulatory agencies of each country/region. Ipilimumab, for example, was approved for use in the USA by the FDA in 2011. That same year the European Commission also approved the ICI. In Brazil the drug was approved by ANVISA in 2012, while in Japan it was approved in 2015.

Assessing the signs and symptoms presented by patients in all studies analyzed, the most prevalent were headache and fatigue. In the image evaluation (MRI), when described, the most common changes were enlargement and enhancement of the pituitary gland. This statement does not apply to Japan, where the majority of images presented were standard, with no significant changes. As expected, the hormonal hypofunctions most commonly described were ACTH, TSH, and LH/FSH deficiency, in that order.

## Limitations

One of the difficulties found in the present study was regarding the classification of hypophysitis in the studies evaluated. Many of them described adrenal insufficiency as a side effect. Regarding the etiology of these pathologies, whether this adrenal insufficiency was primary or secondary was not specified. Other articles only cited endocrinological effects in general as adverse effects, not referring to which ones were present. Many studies also found fatigue as one of the complaints presented. One of the main and initial symptoms of patients with hypophysitis is known to be weakness. Therefore, it is possible that some of these patients did, in fact, present mild hypophysitis without permanent alteration of the pituitary function and, if this is the case, the pathology may have been underdiagnosed.

## Conclusion

The studies reviewed demonstrated an incidence of hypophysitis that ranged from 0% to 27.59%. With hypopituitarism, the incidence ranged from 0% to 33%, and the highest incidence of cases was recorded in a RCT. It was also verified that combination therapy presents a higher risk of leading to the appearance of pituitary alterations, such as hypophysitis, compared to monotherapy. In RCTs, for example, the incidence of hypophysitis ranged from 1.61% to 25.71% with combination therapy, and from 0% to 16.21% in monotherapy. The most common hormonal changes were those of the corticotrophic, thyrotrophic and gonadotrophic axes. Pituitary enlargement and enhancement were the main findings in MRI. The main symptoms presented by patients with hypophysitis were fatigue and headache, and the highest incidence of hypophysitis was found in elderly men.

## Data availability statement

The raw data supporting the conclusions of this article will be made available by the authors, without undue reservation.

## Author contributions

JJ, LN, and MO contributed to conception and design of the study. JJ organized the database and performed the statistical analysis. JJ wrote the first draft of the manuscript. LV, AM, MO and LN wrote sections of the manuscript. All authors contributed to the article and approved the submitted version.
